# Attitudes of medical students towards abortion and their willingness to perform abortion: meta-analysis

**DOI:** 10.4314/ahs.v24i3.28

**Published:** 2024-09

**Authors:** Jian-xin Hu, Rui Chang, Jian-qing Du, Min He

**Affiliations:** 1 School of Nursing and Health, Xi'an Innovation College, Yan'an University, Xi'an, Shannxi 710100, China

**Keywords:** Abortion, medical students, attitudes, opinion

## Abstract

**Background and Objectives:**

This study evaluates the opinion of medical students about abortion and their willingness to perform the abortion.

**Methods:**

After a systematic review, meta-analyses of proportions were performed to achieve percent estimates of medical students' opinions about abortion and willingness to perform the abortion.

**Results:**

15 studies appraising 6341 medical students were included. Most medical students opined that abortion should be provided if the mother's life is threatened (89%), in case of rape (84%), if the mother's mental health is affected (79%), if the fetus is seriously defective (73%); and on mother's request (37%). Medical students informed that they would perform abortion if the mother's life is threatened (87%); in case of rape (77%); if the fetus is seriously defective (78%); if the mother's mental health is threatened (65%); in case of teenage pregnancy (51%); on mother's request (25%); and if court rules (19%). Religiosity was associated with significantly lower proabortion (favoring legalization of abortion) attitudes of medical students (OR: 0.10 [95% CI: 0.04, 0.24]; p<0.00001) but longer duration of medical education was associated with higher proabortion attitudes (OR: 1.75 [95% CI: 1.42, 2.14]; p<0.00001).

**Conclusion:**

Attitudes of medical students towards abortion are generally ambivalent where the majority opine that abortion should be performed under certain circumstances.

## Introduction

The process of removal of the fetus from the womb resulting in the termination of pregnancy is called abortion. It can be a miscarriage if happens spontaneously or induced abortion if artificially performed for therapeutic or personal reasons^1^. The method of abortion depends on fetal age and clinical characteristics of the pregnancy. Often pharmacological agents are used but instrumental/surgical abortion is also common. Abortion may also be complicated by infections, hemorrhage, tissue injuries, uterine puncture, fertility loss, and psychological/psychiatric problems^2^. Unlike most obstetric/gynecological treatments, abortion is not usually provided in hospitals, which also causes its abstinence from medical education^3^.

Worldwide, over 73 million induced abortions are performed annually, and 3 out of 10 of all pregnancies terminate as induced abortions. In developing countries, unsafe abortions are common and approximately 7 million women are hospitalized annually after an unsafe abortion. Annually, 4.7% to 13.2% of women die because of unsafe abortion and rates are much higher for developing countries^4^. The rate of abortion has declined in developed countries where over 90% of all abortions remain complicated, whereas only 45% of abortions remain safe in developing countries. Over 85% of all abortions occur in the developing world where the unintended pregnancy rate is 33%. Worldwide, 40% of women are facing laws that strongly restrict abortion and 26% of women (mainly in the developing world) live with laws that do not allow abortion altogether or permit it in case of threat to mother's life only^5^,^6^.

Attitudes towards abortion are generally classified as either prochoice or prolife. Prochoice respondents consider that women have the right to have control over their bodies whereas prolife respondents believe that the fetus is a live sentient body that has the potential to become a human so abortion should be a prohibited act^7^. However, usually, the response of people is rather ambivalent which tends to deviate from either prolife or prochoice towards conditional approval of abortion^8^. In a survey conducted in 2022, 61% of adult Americans opined that abortion should be legal in all or most cases whereas 37% opined that abortion should be illegal in all or most cases^9^.

Physicians have important roles not only as abortion providers but also through good referrals and facilitations to abortion providers. However, this may be hampered by the prejudices associated with abortion care which have an adverse effect of exclusion of abortion care from medical education^3^,^10^. Even though abortion is common among reproductive women, medical institutions usually do not offer training opportunities for abortion care^11^,^12^. A need to understand the attitudes of medical students toward abortion stems from the fact that they would have to play a role in determining the state of accessibility to abortion in the future. We have undertaken a systematic review of this subject and have performed a meta-analysis of quantitative responses depicting the attitudes of medical students about abortion and their involvement in abortion care.

## Methods

### Inclusion and exclusion criteria

Studies were included if they appraised medical students for seeking their attitudes towards abortion in either cross-sectional or longitudinal design and reported the opinions of medical students about abortion and/or their willingness to perform abortion. Studies were excluded if a) appraised physicians on training, nursing students/trainees, or midwifery students/trainees; b) appraised university students studying in related health disciplines; c) appraised knowledge of abortion or its legal aspects; d) reported qualitative information only.

### Literature search

A systematic literature search was conducted for the identification of study reports published before February 2020. We searched Google Scholar, Ovid SP, PubMed, Science Direct, and Web of Science databases to identify and retrieve required research articles reporting the outcomes of a survey conducted to appraise the opinions of medical students about abortion. Relevant medical subject headings and keywords were used in combinations. These included: abortion, termination of pregnancy, induced, medical, health, students, attitudes, opinion, perception, appraisal, and survey. Additionally, software suggested articles and reference lists (bibliographies) of important research articles were also screened for relevant studies.

### Data extraction, synthesis, and statistical analysis

Demographic, study design, instrument, survey, and response data were extracted from research articles of included studies. Meta-analyses of proportions were performed with Stata software (Stata Corporation, Texas, USA) using binomial response data of appraisals reported by the individual studies. Freeman Tukey's double arcsine transformation of proportions was incorporated using the exact binomial method for variance stabilization. Appraising domains of interest were: a) Under which circumstances abortion should be permitted, and b) under which circumstances you are willing to perform the abortion. Items of interest for both these domains were: a) if the mother's life is threatened; b) in case of rape; c) if the fetus is seriously defective; d) if the mother's mental health is threatened; e) in case of teenage pregnancy; f) if court rules; and f) on mother's request. For examining the possible impacts of religiosity, gender, and length of medical education, the meta-analyses of odds ratios (OR) were performed with Cochrane's Review Manager software (Nordic Cochrane Centre, Cochrane Collaboration, Copenhagen, Denmark). Statistical heterogeneity (inconsistency of outcomes between studies) was estimated with the I2 index.

## Results

**Figure 1:**
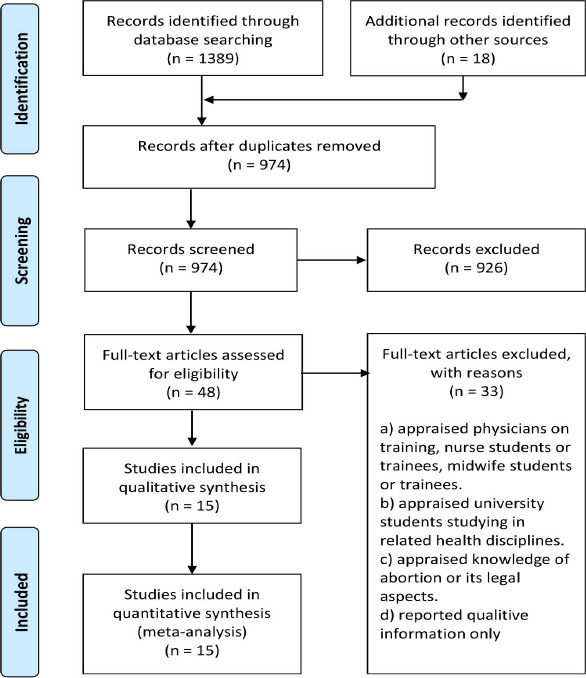
A flowchart showing the process of study selection process after literature search

Fifteen studies13-27 appraising 6341 medical students were included in this systematic review and meta-analysis (Figure 1). The mode of the survey was online in 3 studies, postage in 2 studies, and institution-based in 10 studies. All studies were cross-sectional in design. These studies were conducted in the USA (3), UK (2), Argentina (1), Ireland (2), South Africa (2), Bosnia & Herzegovina (1), Canada (1), Thailand (1), Turkey (1), and West Indies (1). The percentage of female respondents in this population was 56% [95% confidence interval (CI): 48, 63]. The percentage of religious believers was 67% [95% CI: 54, 79]. Characteristics of the included studies are given in Table S1.

**Table S1:** Characteristics of the included studies

Study	n	Country	Mode of survey	Age (years)	% Females	% Unmarried	% Religious
Bennett 2018	315	USA	Online	25.4±3.0	52.4	76.5	77.4
Buga 2002	246	South Africa	Self-administered		55.3	95.3	99.6
Dans 1992	658	USA	Self-administered				
Fitzgerald 2013	169	Ireland	Online	88% <30	55	84	
Gleeson 2008	100	UK	Self-administered	19.2±1.29	63		69
Mathews 2020	1404	West Indies	Online	22.7±2.9	42.5	62	75.5
Merrill 1993	187	USA	Self-administered				
Myran 2015	308	Canada	Postal	23.8±0.85	50		
O'Grady 2015	525	Ireland	Self-administered	22.2±2.9	58		61
Ozmen 2018	65	Turkey	Self-administered	21–24	69		37
Provenzano-Castro 2016 *	760	Argentina	Self-administered	81% 18–24	73.8	95.8	
Steele 2009	145	UK/Norway	Self-administered		69		27
Trninic 2017	148	Bosnia/Herzegovina	Self-administered				92
Varakamin 1977	318	Thailand	Postal	85% 22–24	22		
Wheeler 2012	1308	South Africa	Self-administered		60	51.7	67.3

* In Provenzano-Castro 2016 study, sample size included 445 medical students, 142 nursing students, 50 midwifery students and 123 students from other health disciplines. However, in meta-analysis only data of medical students are used.

**Figure 2:**
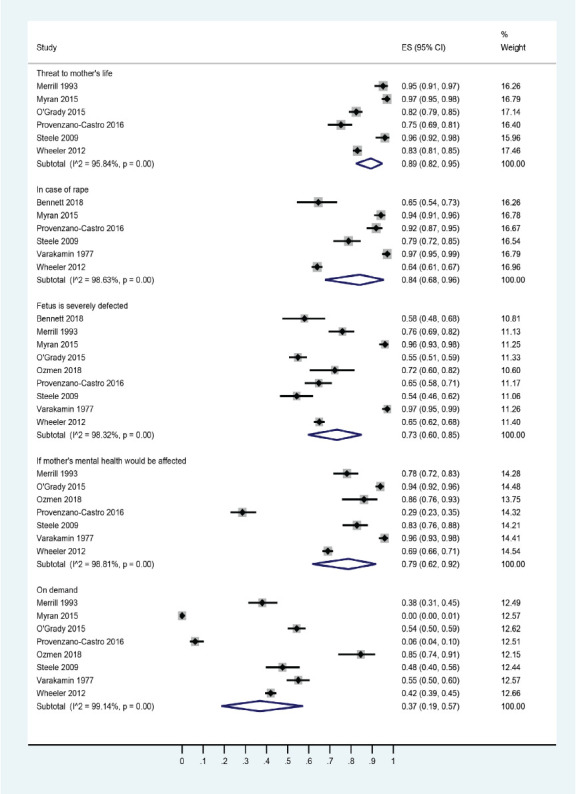
A forest graph showing the pooled estimates of the responses of medical students against the appraisal ‘under which circumstances abortion should be allowed’

Endorsing responses of medical students to question ‘under which circumstances should abortion be permissible’ were as follows: a) if the mother's life is threatened (89% [95% CI: 82, 95]); b) in case of rape (84% [95% CI: 68, 96]); c) if the mother's mental health is threatened (79% [95% CI: 62, 92]); d) if the fetus is seriously defective (73% [95% CI: 60, 85]); and e) on mother's request (37% [95% CI: 19, 57]; Figure 2).

**Figure 3:**
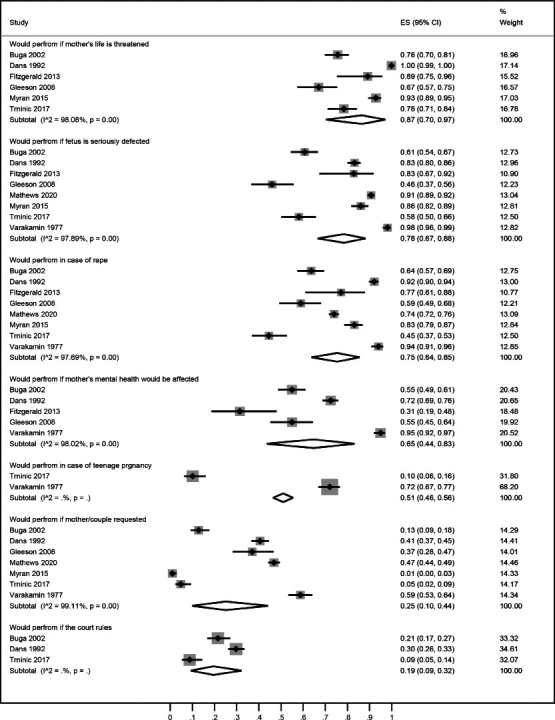
A forest graph showing the pooled estimates of the responses of medical students against the appraisal ‘under which circumstances they will perform abortion’

Endorsing responses of medical students to question ‘under which circumstances they would perform abortion’ were as follows: a) if the mother's life is threatened (87% [95% CI: 70, 97]); b) in case of rape (75% [95% CI: 64, 85]); c) if the fetus is seriously defective (78% [95% CI: 67, 88]); d) if the mother's mental health is threatened (65% [95% CI: 44, 83]); e) in case of teenage pregnancy (51% [95% CI: 46, 56]); f) on mother's request (25% [95% CI: 10, 44]); and g) if court rules (19% [95% CI: 9, 32]) (Figure 3).

**Figure S1:**
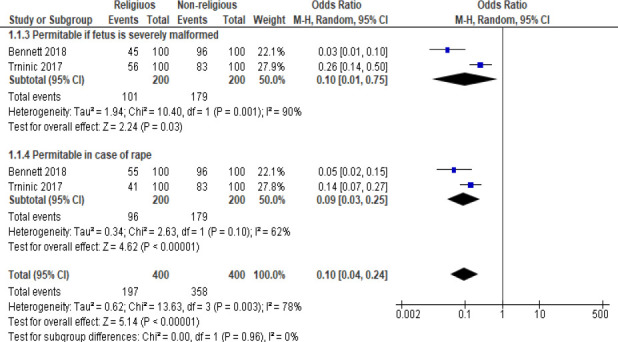
A forest graph showing the effect of religion on medical students' responses

**Figure S2:**
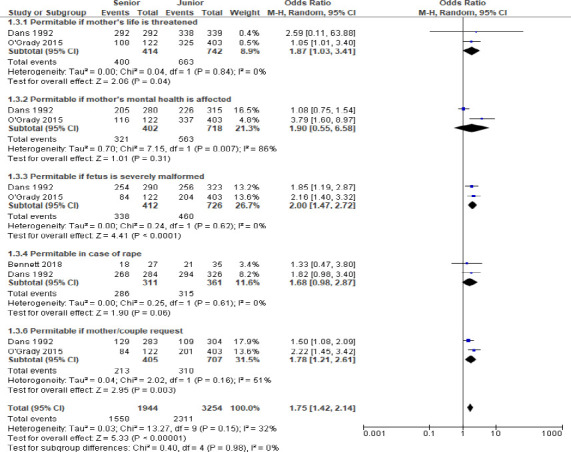
A forest graph showing the effect of duration of medical education on medical students' responses

Fewer data were available to study the effects of gender, religiosity, and medical education on the attitudes of medical students about abortion. Within the available data, religiosity was associated with significantly lower proabortion (favoring legalization of abortion) attitudes of medical students (OR: 0.10 [95% CI: 0.04, 0.24]; p<0.00001; Figure S1). On the other hand, a longer duration of medical education was associated with higher proabortion attitudes (OR: 1.75 [95% CI: 1.42, 2.14]; p<0.00001; Figure S2).

## Discussion

This meta-analysis found that the opinions of medical students towards abortion are ambivalent where a considerably high proportion of medical students believe that abortion should be provided under some circumstances and were willing to provide abortion themselves under such circumstances including threat to mother's life or health, fetal abnormality, and for rape victims.

Generally, physicians favor abortion provision and are usually prochoice. Only 10% of Irish general practitioners are against abortion under any circumstance, 25% allow it under limited circumstances, and 51% support abortion for all women upon request^28^. A study conducted in the UK found that 82% of general practitioners favor the provision of abortion to all women^29^. In the present study, most medical students were willing to perform or refer to abortion under some circumstances but a smaller proportion of medical students favored abortion upon any request. In comparison with experienced physicians, young graduates are found to be less interested in providing abortion^30^. Thus, a shortage of abortion providers can be foreseen even in countries with better legislation and facilities which can create difficulties for women seeking an abortion. Such a shortage has already been observed in some areas of the USA and Canada^19^.

Religiosity is a strong preventing factor towards legalization of abortion. Frequent church attendance is found to be associated with higher antiabortion attitudes even after controlling for several confounders^31^,^32^. In the present meta-analysis, the percentage of religious believers was approximately 67% [95% CI: 34, 84. Although, numerical data were less available to study the effect of religiosity in the present study, a meta-analysis of 2 studies found it to be associated with antiabortion views. Moreover, some other included studies also noted an effect of religiosity on the attitudes of medical students towards antiabortion drive. One of the included studies in which students from 2 different medical institutions were appraised found that in one institution, 48% of the respondents had no religious affiliation and 78.2% were proabortion, whereas in the other institution, proportion of respondents with no religious affiliation was 4.7% and 14.3% had proabortion attitudes^24^. Wheeler et al.^27^, found that Christians and Muslims were less likely to accept abortion for any reason morally than Hindus and Jews or atheists. Many other studies have also found associations between attitudes toward abortion and religiosity^33^-^35^.

Social environment may also have a role in determining attitudes toward abortion. It has been found that medical students whose perceptions about abortion were associated with social opposition were less likely to show willingness to perform the abortion in future practice in comparison with students whose social perceptions were not antiabortion^20^. Students with sexual experience are found to be more liberal towards their abortion attitudes^36^. A systematic review found that abortion was more permissible for married adolescents in comparison with unmarried adolescents^37^. On the other hand, a review found that abortion decision-makers were most often the male partners^38^. Studies show that abortion attitudes are not usually affected by the gender of respondents^32^. Among the included studies of this meta-analysis, one study found no significant gender differences in opinions^13^, whereas another found statistically significant gender differences in the attitudes of medical students in several appraisal items^21^.

An important finding of the present study is that senior medical students were more proabortion than junior students which shows that during medical education the attitudes of medical students transform. However, the precise role of medical education in this transformation remains obscured as other factors may play a role. A study found that graduates had relatively higher conscientiousness, confidence, self-control, morality, and empathy in comparison with medical school entrants^39^. Moreover, exposure to related training may also have a role in changing attitudes. It has been reported that formal and informal education about abortion was associated with intention of family medicine residents to provide abortion^40^ and that physicians who receive abortion training during residency are more likely to perform abortions afterward^41^. A study found that medical students became active supporters from passive supporters of abortion after observing the experiences of women who decided for abortions under difficult circumstances^11^. Similar outcomes are also reported for nursing where nurses were found to be more proabortion in comparison with nursing students^42^.

An important limitation of the present study is that meta-analyses observed substantial variations in the responses of medical students which were also evident from statistical heterogeneity (I2≥95%). It was not possible to evaluate the sources of heterogeneity in the outcomes because of less availability of data, especially to study the impacts of gender, religiosity, and social and socioeconomic background on the outcomes. Appraising instruments of the individual studies were also varying to some extent in terms of terminology and comprehension which could have an impact on students' responses and therefore might have added heterogeneity to meta-analyses. Although, in most of these studies, the survey instruments were self-administered by the researchers, a few studies conducted mail-based/online surveys to which response was variable which might have impacted the overall outcomes to some extent. Included studies were conducted mainly in developed countries where the incidence of abortion is lower than in developing countries. This restricts the global applicability of the present study.

There is a considerable variation in the opinions of medical students towards abortion and in their willingness to provide abortion. Overall, an ambivalent response has been achieved herein where a considerably high proportion of medical students was in favor of providing abortion under some circumstances including the threat to mother's life or health, rape-caused pregnancy, and serious fetal defect. First-year medical students were less in favor of abortion than final-year students. Gender appears to have less impact on the opinions, but religiosity may have significant impact on the attitudes of medical students.
